# Associations among smoking, *MGMT* hypermethylation, *TP53*-mutations, and relapse in head and neck squamous cell carcinoma

**DOI:** 10.1371/journal.pone.0231932

**Published:** 2020-04-23

**Authors:** Shinichi Matsuda, Aki Mafune, Nagisa Kohda, Takanori Hama, Mitsuyoshi Urashima

**Affiliations:** 1 Division of Molecular Epidemiology, The Jikei University School of Medicine, Tokyo, Japan; 2 Real World Data Science Department, Chugai Pharmaceutical Co. Ltd., Tokyo, Japan; 3 Division of Kidney and Hypertension, Department of Internal Medicine, The Jikei University School of Medicine, Tokyo, Japan; 4 Department of Oto-Rhino-laryngology, The Jikei University School of Medicine, Tokyo, Japan; University of California, San Francisco, UNITED STATES

## Abstract

**Background:**

Epigenetic silencing of the O^6^-methylguanine-DNA methyltransferase (*MGMT*) DNA repair enzyme via promoter hypermethylation (hm*MGMT*) may increase mutations in the *TP53* oncosuppressor gene and contribute to carcinogenesis. The effects of smoking, which is a risk factor for head and neck squamous cell carcinoma (HNSCC), were investigated to determine whether they up- or down-regulate hm*MGMT*. Additionally, the impact of hm*MGMT* and disruptive *TP53*-mutations on relapse was investigated in patients with HNSCC.

**Methods:**

This study included 164 patients with HNSCC who were negative for both *p16* protein expression and human papilloma virus infection. The association of smoking and hm*MGMT* was investigated using multiple logistic regression analysis. Competing risk regression was used to evaluate the effects of hm*MGMT* and *TP53*-mutations in exon 2 to 11 on relapse of HNSCC.

**Results:**

hm*MGMT* was observed in 84% of the 164 patients. *TP53*-mutations, specifically, G:C>A:T transition, were more frequent in patients with hm*MGMT* (32%) than in those without hm*MGMT* (8%). The frequency of disruptive *TP53*-mutations was not significantly different between groups. Compared with nonsmoking, heavy smoking of 20 pack-years or more was significantly associated with decreased hm*MGMT* (adjusted odds ratio, 0.08; 95% CI, 0.01 to 0.56; *P* = 0.01). Patients who had both hm*MGMT* and disruptive *TP53-*mutations showed a significantly higher relapse rate than all other patients (subdistribution hazard ratio, 1.77; 95% CI, 1.07 to 2.92; *P* = 0.026).

**Conclusions:**

It was found that hm*MGMT* was suppressed by heavy smoking, and hm*MGMT* combined with disruptive *TP53-*mutations may indicate a poor prognosis in patients with HNSCC.

## Introduction

Head and neck squamous cell carcinoma (HNSCC) is one of the most common cancers worldwide. Approximately 550,000 cases of HNSCC are newly diagnosed each year in the world, and only 40–50% of patients with HNSCC survive for 5 years [1]. It is well known that HNSCC is a multifactorial disease with contributing etiologies including tobacco smoking, alcohol consumption, and infection with the human papillomavirus (HPV) [2, 3]. In addition, some reports have shown the importance of epigenetic mechanisms in the development and progression of HNSCC and other cancers [4, 5].

O^6^-methylguanine DNA methyltransferase (*MGMT*) is one of the DNA repair enzymes that protects genes from mutations by directly removing cytotoxic alkyl adducts from the O^6^ position of guanine [6]. The expressions of *MGMT* RNA and protein are decreased by methylation of a CpG island in its promoter region [7, 8]. Thus, aberrant hypermethylation of the *MGMT* promoter region (hm*MGMT*) may hamper its DNA repair function, allowing mutations of G:C>A:T transition in *TP53*, as well as other carcinogenic genes, in various cancers [9–12].

Recently, genotoxic stressors such as tobacco smoking have been investigated with regards to their possible involvement in the regulation of *MGMT* [13]. Several studies have demonstrated an increase in *MGMT* activity/expression in the normal/tumor tissue of smokers compared to non-smokers, suggesting the possible role of tobacco smoking in regulating *MGMT* protein expression in the tissue [14–16]. However, studies of the effect of smoking on hypermethylation of the *MGMT* promoter (hm*MGMT*) reported conflicting results. In HNSCC, there has been only one study that showed that hm*MGMT* was unchanged by smoking [17]. Regarding other types of cancers, hm*MGMT* was reported to be upregulated in lung adenocarcinoma [18], downregulated in non-small cell lung cancer [19], or unchanged in non-small cell lung cancer [20] by smoking. One possible explanation for this discrepancy may be the differences in the analysis methods used. For example, one study defined smoking status as a binary characteristic (i.e. nonsmoker and smoker), whereas the other study defined smoking status based on the degree of smoking (i.e. pack-years). In addition, because the numbers of patients in the previous studies were relatively small, the association between smoking and hm*MGMT* was often evaluated by means of a simple chi-squared test, or evaluated by adjustment for only limited confounders. Thus, there were no studies that considered enough confounders, such as cancer stage, primary site of cancer, differentiation, and degree of alcohol consumption. In the present study, the aim was to clarify whether smoking enhances or suppresses hm*MGMT* in HNSCC by performing multivariate adjustment for potential confounders. Additionally, the effects of hm*MGMT* and *TP53* mutations on relapse in patients with HNSCC were analyzed.

## Materials and methods

### Ethics statement

The study protocol was reviewed and approved by the Ethics Committee for Biomedical Research of the Jikei Institutional Review Board. Written, informed consent was obtained from all patients enrolled in the study.

### Study design

This study was a post hoc analysis of our prospective cohort study [21, 22], which was conducted at Jikei University Hospital from March 2006 to November 2012. The entire process of study design, data monitoring, and analyses was performed at the Division of Molecular Epidemiology. Eligible participants were Japanese patients with HNSCC (oropharyngeal, hypopharyngeal, laryngeal, oral cavity, and sinonasal cancer) aged 20 years and over, who had newly diagnosed or recurrent disease, and who had surgical resection with curative intent before chemoradiotherapy. Clinical information was obtained from clinical and surgical charts. The tumor node metastasis (TNM) classification and cancer stages were determined according to the 6th Union for International Cancer Control TNM classification and stage groupings.

Based on the above cohort, this study excluded patients with high-risk HPV infections (16/18/31/33/35/52b/58) and patients who tested positive for p16, because this subpopulation is known to have a different etiology and pathogenesis from smoking/alcohol-induced HNSCC [1]. HPV infection was detected using multiplex polymerase chain reaction (PCR) with the TaKaRa Human Papillomavirus Typing Set #6603 following the manufacturer’s protocol (Takara Bio Inc., Shiga, Japan). Positive p16 expression, which was defined as strong and diffuse nuclear and cytoplasmic staining in at least 70% of tumor cells was detected by immunohistochemistry using a rabbit monoclonal antibody to p16 (Anti-CDKN2A/p16INK4a antibody [EPR1473]): Abcam plc, Science Park, Cambridge, England).

### Smoking and alcohol consumption

Patients were divided into the following three groups based on smoking status prior to diagnosis of HNSCC: (1) nonsmokers, defined as patients who had never used tobacco or had stopped using tobacco for more than 20 years; (2) moderate smokers, defined as current or past smokers who smoked less than 20 pack-years within the last 20 years; and (3) heavy smokers, defined as current or past smokers who had smoked 20 pack-years or more within the last 20 years. This definition of heavy smokers is consistent with the study that reported that a cumulative dose corresponding to 20 cigarettes per day over 10–20 years or 10–20 pack-years is associated with a clinically relevant increase in morbidity [23, 24].

Patients were divided into the following three categories based on average daily alcohol consumption during the 20 years preceding diagnosis of HNSCC: (1) non-drinkers, defined as non-drinkers or light drinkers who consumed less than one drink per day; (2) moderate drinkers, defined as drinkers who consumed at least one but less than two drinks per day; and (3) heavy drinkers, defined as drinkers who consumed two or more drinks per day. One drink was defined as containing approximately 10 g of alcohol, which is equal to 30 mL of hard liquor, 100 mL of wine containing 12% alcohol, or 360 mL of beer.

### Samples

With each patient’s consent, tumor and margin samples from the primary site, but not metastatic sites, were collected. These samples were rapidly frozen and stored at -80 °C after excision. The cancer tissue was divided into two specimens: one for pathological confirmation in which the sample was composed of >70% cancer cells and the other for DNA extraction. DNA was extracted and purified using the QIAamp DNA Micro Kit 50 (QIAGEN, Tokyo, Japan), and the DNA concentration of the samples was measured using NanoVue plus (General Electric Healthcare Japan, Tokyo, Japan). Samples were then frozen at -80 °C until use.

### Methylation-specific PCR for detection of hypermethylation of the *MGMT* promoter

The methylation-specific polymerase chain reaction (MSP) was used to distinguish between hm*MGMT* and non-hm*MGMT*. Briefly, DNA samples extracted from tumor tissues were treated with bisulfite using MethylEasy Xceed (Takara Bio Inc.) according to the manufacturer’s protocol. Subsequently, MSP was carried out using the EpiScope MSP kit (Takara Bio Inc.). The primers used for PCR were described previously [25]. PCR was carried out in a 50-μL volume containing 4 μg of bisulfite-treated DNA, 25 μL of 2xMSP buffer, 1.2 μL of MSP enzyme, 0.5 μL of 100xSYBR Green I, 16.3 μL of nuclease-free water, and 1.5 μL of each of the two primers. The reaction was incubated at 95 °C for 30 sec, followed by 35 cycles at 98 °C for 5 sec, 55 °C for 30 sec, and 72 °C for 1 min, with a final incubation step at 16 °C.

### Analysis of *TP53*-mutation

Exons 2 to 11 of the *TP53*-gene were amplified using PCR with purchased primers following the manufacturer’s protocol (Nippon Gene Co. Ltd., Tokyo, Japan), cloned, and then sequenced using the ABI PRISM 3700 Genetic Analyzer (Applied Biosystems, Foster City, CA). Disruptive *TP53*-mutations are defined as non-conservative mutations located inside the key DNA-binding domain (L2-L3 region) or stop codons in any region [26]. The missense changes (V31I, P36P, P47S, P72R, R72R, R158R, R213R, V217M, P222P, T312S, and G360A) reported as single nucleotide polymorphisms [27] were not included in the total *TP53* mutations.

### Statistical analysis

Patients’ characteristics and *TP53-*mutation status were compared between the hm*MGMT* and non-hm*MGMT* groups using Pearson’s chi-squared test, Student’s *t*-test, and the Mann-Whitney test, as appropriate. To clarify whether smoking enhances or suppresses hm*MGMT*, odds ratios (ORs) and 95% confidence intervals (CIs) were estimated using the following multivariate logistic regression models: Model I was adjusted only by smoking status (nonsmoker, moderate smoker, heavy smoker); Model II was adjusted by age, sex, alcohol consumption (none, moderate, heavy), and primary site of tumor, in addition to the variables in model I; Model III was adjusted by tumor stage (stage I to IV), in addition to the variables in model II; Model IV was adjusted by tumor cell differentiation status (well, moderately, poorly differentiated), in addition to the variables in model III.

In survival analyses, the time from surgery to relapse was used to calculate the relapse-free ratio. To evaluate the effects of hm*MGMT* and *TP53*-mutations on the relapse of HNSCC, cumulative incidence functions (CIFs) were applied by considering patients’ death by causes other than the relapse as the competing risk. Competing risk regression was performed by the Fine and Gray subdistribution hazard model [28]. A value of *P* < 0.05 was considered significant. All statistical analyses were performed using STATA 14.2 (STATA Corp., College Station, TX).

## Results

### Associations among hm*MGMT*, *TP53*-mutations, and patient characteristics

A total of 164 patients who were negative for both HPV gene and p16 were analyzed. The patients’ characteristics stratified by the methylation status of *MGMT* (hm*MGMT vs*. non-hm*MGMT*) are summarized in Table 1. Eighty-four percent of the study population was classified as hm*MGMT*. There were no significant differences in age, sex, primary site of tumor, differentiation, smoking, or drinking status between hm*MGMT* and non-hm*MGMT* patients. On the other hand, cancer stages were more advanced in hm*MGMT* patients than in non-hm*MGMT* patients (*P* < 0.001).

**Table 1:** Patients’ characteristics according to the methylation status of *MGMT*.

Variable	All patients (n = 164)	Patients with hypermethylated MGMT (n = 138: 84%)	Patients with non-hypermethylated MGMT (n = 26: 16%)	*P* value
Age, years; mean ± SD	63.7 ± 10.8	64.1 ± 10.8	61.4 ± 10.4	0.24 ^a^
Men, no. (%)	132 (80)	113 (82)	19 (73)	0.30 ^b^
Primary site of tumor, no. (%)				0.38 ^b^
Oropharynx	29 (18)	26 (19)	3 (12)	
Hypopharynx	51 (31)	45 (33)	6 (23)	
Larynx	22 (13)	16 (12)	6 (23)	
Oral cavity	50 (30)	42 (30)	8 (31)	
Sinonasal	12 (7)	9 (7)	3 (12)	
Differentiation, no. (%)				0.52 ^b^
Well differentiated	49 (31)	42 (32)	7 (28)	
Moderately differentiated	78 (49)	67 (50)	11 (44)	
Poorly differentiated	31 (20)	24 (18)	7 (28)	
Stages, no. (%)				<0.001 ^b^
I	11 (7)	5 (4)	6 (23)	
II	32 (20)	30 (22)	2 (8)	
III	36 (22)	26 (19)	10 (38)	
	84 (52)	76 (55)	8 (31)	
Smoking status, no. (%)				0.32 ^b^
Non-smoker	44 (27)	39 (28)	5 (20)	
Moderate smoker	16 (10)	15 (11)	1 (4)	
Heavy smoker	102 (63)	83 (61)	19 (76)	
Drinking status, no. (%)				0.15 ^b^
Non-drinker	58 (35)	46 (33)	12 (46)	
Moderate drinker	59 (36)	54 (39)	5 (19)	
Heavy drinker	47 (29)	38 (28)	9 (35)	

Percentages may not sum to 100% because of rounding.

^a^ *P* value was calculated using Student’s *t-*test.

^**b**^ *P* value was calculated using Pearson’s chi-squared test.

Frequencies of *TP53*-mutation spectra were compared between the hm*MGMT* and non-hm*MGMT* groups. The proportion of patients who had at least one *TP53*-mutation was significantly higher in the hm*MGMT* (76%) than in the non-hm*MGMT* (50%) group (*P* = 0.007). Patients with hm*MGMT* tended to have a greater number of *TP53*-mutations per patient than patients with non-hm*MGMT* (*P* = 0.003). Regarding the type of *TP53* mutation, G:C>A:T transition was significantly more common in hm*MGMT* (32%) than in non-hm*MGMT* (8%) (*P* = 0.012). Frequencies of disruptive *TP53*-mutations were 26% and 15%, respectively, and they were not significantly different (*P* = 0.244). No other type of mutation was significantly different between hm*MGMT* and non-hm*MGMT*.

### Association between smoking and hm*MGMT*

To determine whether smoking is associated with hm*MGMT*, multivariate logistic regression analyses were performed (Table 2). In Model I, smoking status did not show significant associations with hm*MGMT*. In contrast, in models II, III, and IV, heavy smoking was significantly associated with a reduced frequency of hm*MGMT* (in model IV, adjusted OR, 0.08; 95% CI, 0.01 to 0.82; *P* = 0.03).

**Table 2:** Effects of smoking on hm*MGMT* using logistic regression models.

		Adjusted OR	95% CI	*P* value
Model I ^a^	Non-smoker	Reference	-	
	Moderate smoker	1.92	0.21 to 17.9	0.57
	Heavy smoker	0.56	0.19 to 1.61	0.28
Model II ^b^	Non smoker	Reference	-	
	Moderate smoker	1.19	0.09 to 15.7	0.89
	Heavy smoker	0.08	0.01 to 0.56	0.01
Model III ^c^	Non smoker	Reference	-	
	Moderate smoker	1.88	0.09 to 39.4	0.68
	Heavy smoker	0.08	0.01 to 0.66	0.02
Model IV ^d^	Non smoker	Reference	-	
	Moderate smoker	2.87	0.09 to 93.1	0.55
	Heavy smoker	0.08	0.01 to 0.82	0.03

^a^ Model I was adjusted only by non-smoker, moderate smoker, and heavy smoker.

^b^ Model II was adjusted by age, sex, alcohol consumption (non, moderate, and heavy), and primary site of tumor, in addition to the variables used in model I.

^c^ Model III was adjusted by cancer stage I to IV, in addition to the variables used in model II.

^d^ Model IV was adjusted by well, moderately, and poorly differentiated tumor, in addition to the variables used in model III. OR: odds ratio, CI: confidence interval

### Association between hm*MGMT* and relapse

**Fig 1:**
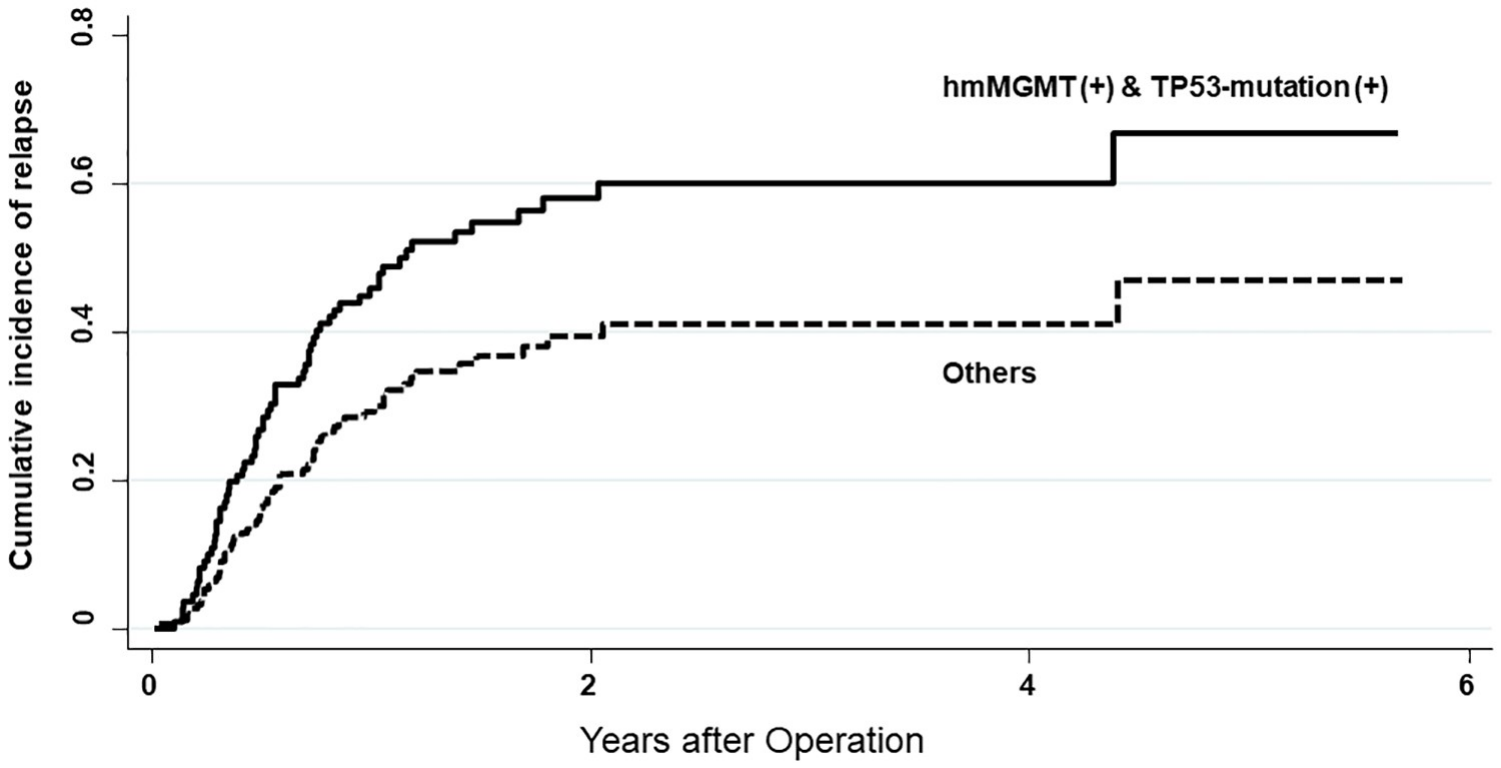
Competing risk regression for relapse of HNSCC.

The impact of hm*MGMT* and disruptive *TP53*-mutations on relapse of HNSCC was investigated by competing risk regression. Patients with hm*MGMT* did not show a significantly higher risk of relapse compared with those without hm*MGMT* (subdistribution hazard ratio [SHR], 1.73; 95% CI, 0.83 to 3.59; *P* = 0.141). In addition, patients with disruptive *TP53*-mutations did not show a significantly higher risk of relapse compared with those without the mutations (SHR, 1.48; 95% CI, 0.89 to 2.45; *P* = 0.129). In contrast, the subgroup of patients who were positive for both hm*MGMT* and disruptive *TP53*-mutations showed a significantly higher risk of relapse than all of the other patients (SHR, 1.77; 95% CI, 1.07 to 2.92; *P* = 0.026) (Fig 1).

## Discussion

This study demonstrated that heavy smoking (20 pack-years and more) was inversely associated with hm*MGMT* after adjusting for possible confounders. The previous studies [17–20] did not adjust for several confounders such as tumor stage and alcohol consumption. In the present study, the degree of smoking (non-, moderate-, heavy smokers) was considered, which has not been previously distinguished but was considered a binary characteristic (never smokers or smokers) in other studies.

Previously, smoking was reported to upregulate *MGMT* protein expression and activity [13], which is consistent with the present result that heavy smoking downregulates the methylation of *MGMT* because this is expected to result in upregulation of *MGMT* RNA and protein expressions. A similar association between lower levels of hm*MGMT* and smoking was reported in colorectal adenoma [29]. In the present study, as a kind of biological defense mechanism, it was speculated that smoking might trigger a variety of gene mutations and simultaneously upregulate *MGMT* expression through demethylation of the *MGMT* promoter region in order to repair G:C>A:T transition. Additional studies are necessary to investigate the reasons for the conflicting results obtained from different types of cancers previously. As of now, there would be possibilities of effects from ethnic diversity or different cancer pathogeneses.

In agreement with previous reports [30, 31], there was a higher frequency of *TP53* mutation in patients with hm*MGMT* than in those with non-hm*MGMT*. Regarding the type of *TP53* mutation, G:C>A:T transition was significantly more common in patients with hm*MGMT* than in those with non-hm*MGMT*. The obtained result is theoretically plausible, since decreased expression of *MGMT* protein through hm*MGMT* allows O^6^-alkylguanine adducts to pair with thymine during DNA replication, resulting in a G:C>A:T transition mutation [9]. The present findings showed that hm*MGMT* is associated with a high frequency of *TP53* mutations, particularly with G:C>A:T transitions in HNSCC.

A previous study showed that the prognostic value of *TP53*-mutation varied by the prediction method used, and Poeta rules, which is a prediction algorithm based on whether the mutation is disruptive or non-disruptive, did not significantly predict the prognosis of HNSCC [32]. In the present study, patients who were positive for hm*MGMT* and disruptive *TP53*-mutations showed a higher relapse rate. hm*MGMT* may allow mutations not only in the *TP53* gene, but also in the genes of other oncosuppressors and oncogenes [33]. Thus, these patients that had both hm*MGMT* and disruptive *TP53*-mutations might have shown a poor prognosis.

This study has several limitations. First, the study included not only patients with newly diagnosed, but also those with recurrent HNSCC. Thus, the previous therapies might have affected the methylation status of *MGMT* in patients with recurrent HNSCC. In addition, because all patients in this study underwent surgery, the proportion of hm*MGMT* obtained from this study may not indicate the proportion in the whole HNSCC population. Second, the effects of primary sites of HNSCC could not be adequately explored due to the limited sample size. Third, most patients in the present study had advanced stage III to IV disease (74%). Therefore, generalization of the present findings to patients in earlier stages would be limited. Last, *MGMT* RNA and protein expressions were not measured. However, several reports have shown a significant correlation between *MGMT* methylation status and its protein expression in patients with HNSCC [31, 34, 35].

In conclusion, hm*MGMT* was suppressed by heavy smoking, and hm*MGMT*, combined with disruptive *TP53-*mutations, may be associated with a poor prognosis in patients with HNSCC.

## Supporting information

S1 Data Data set of the present study. (XLSX)
